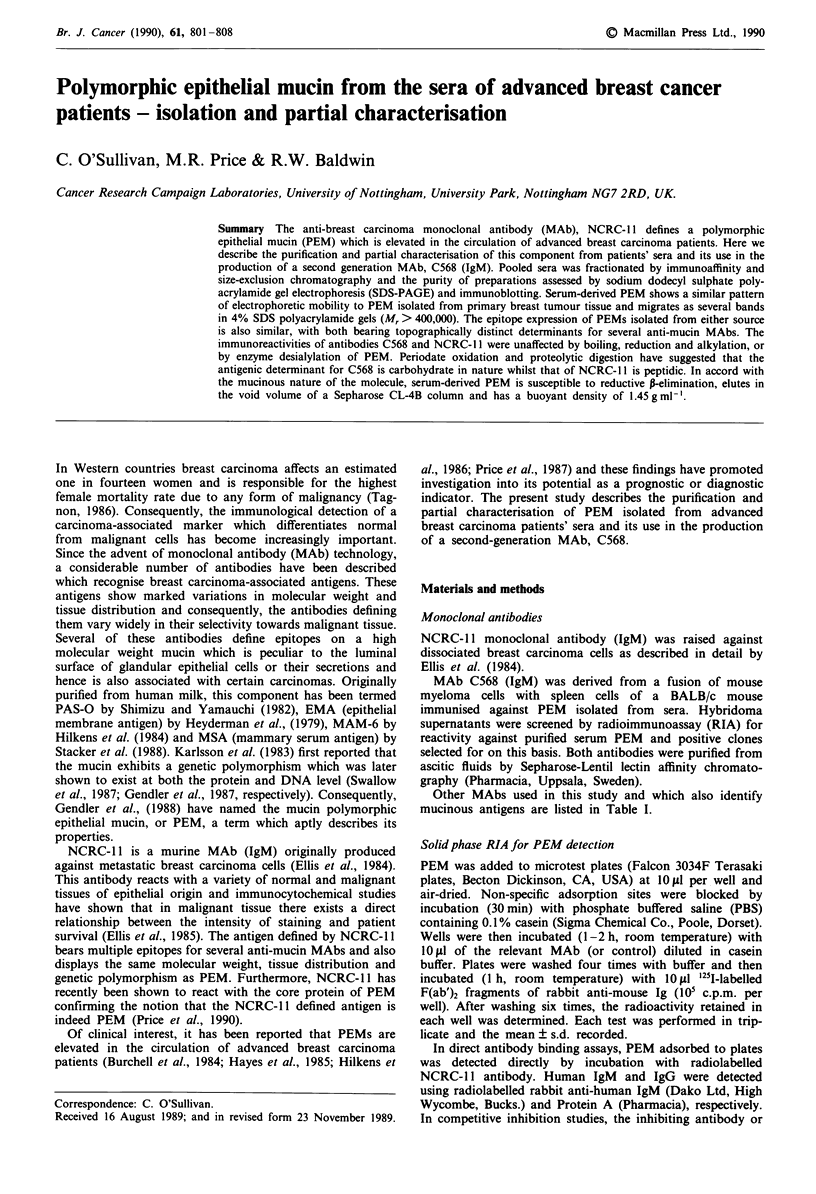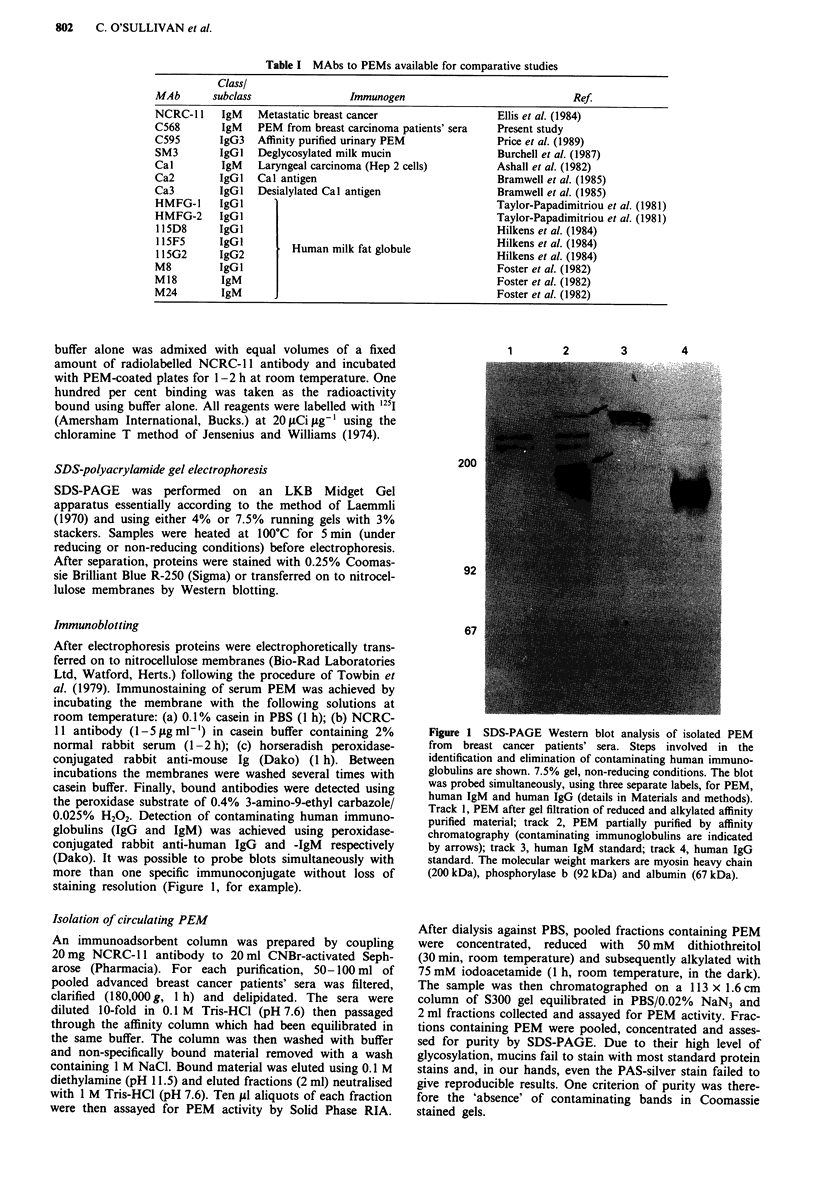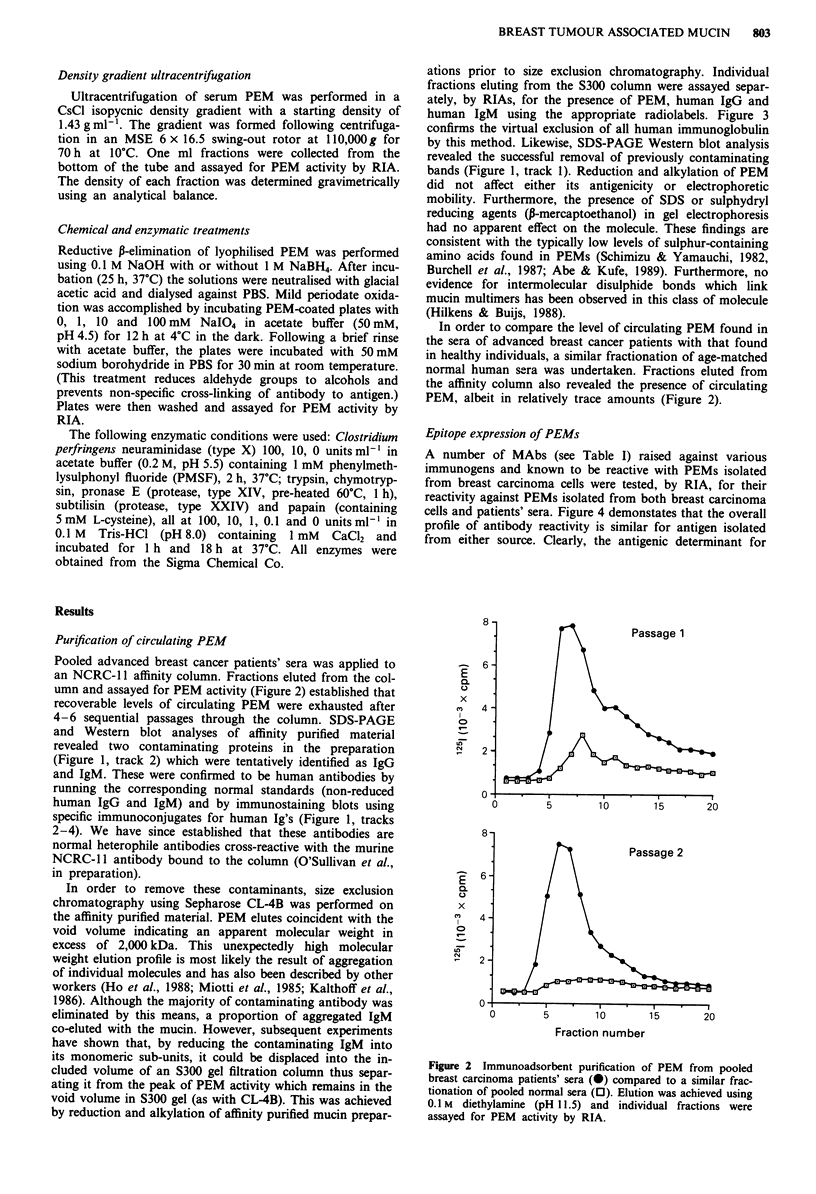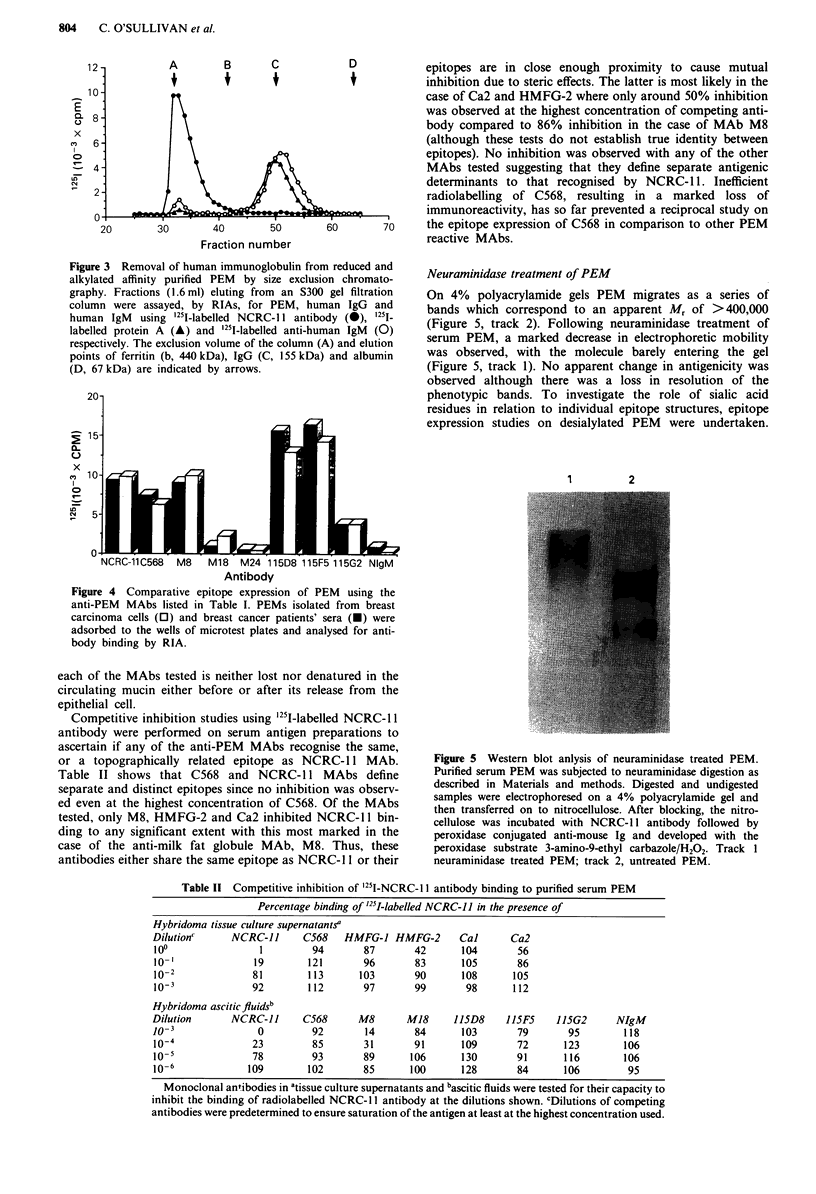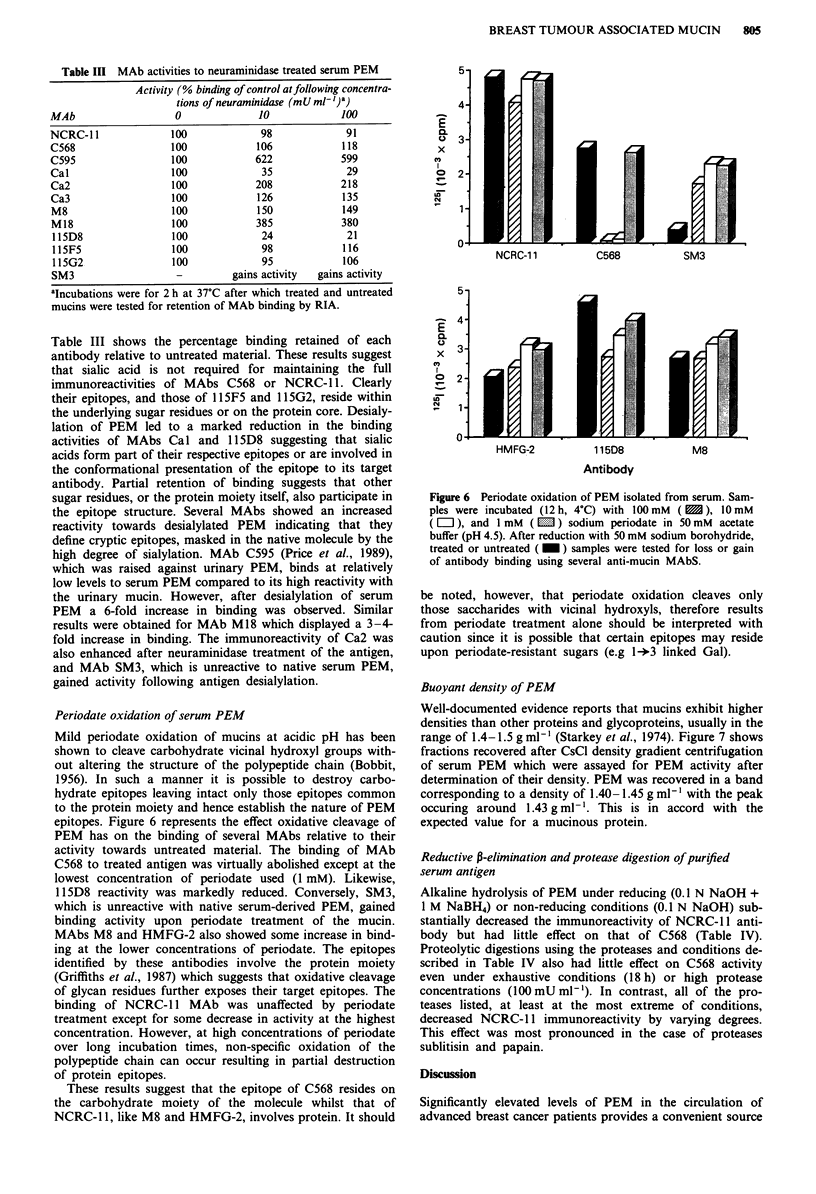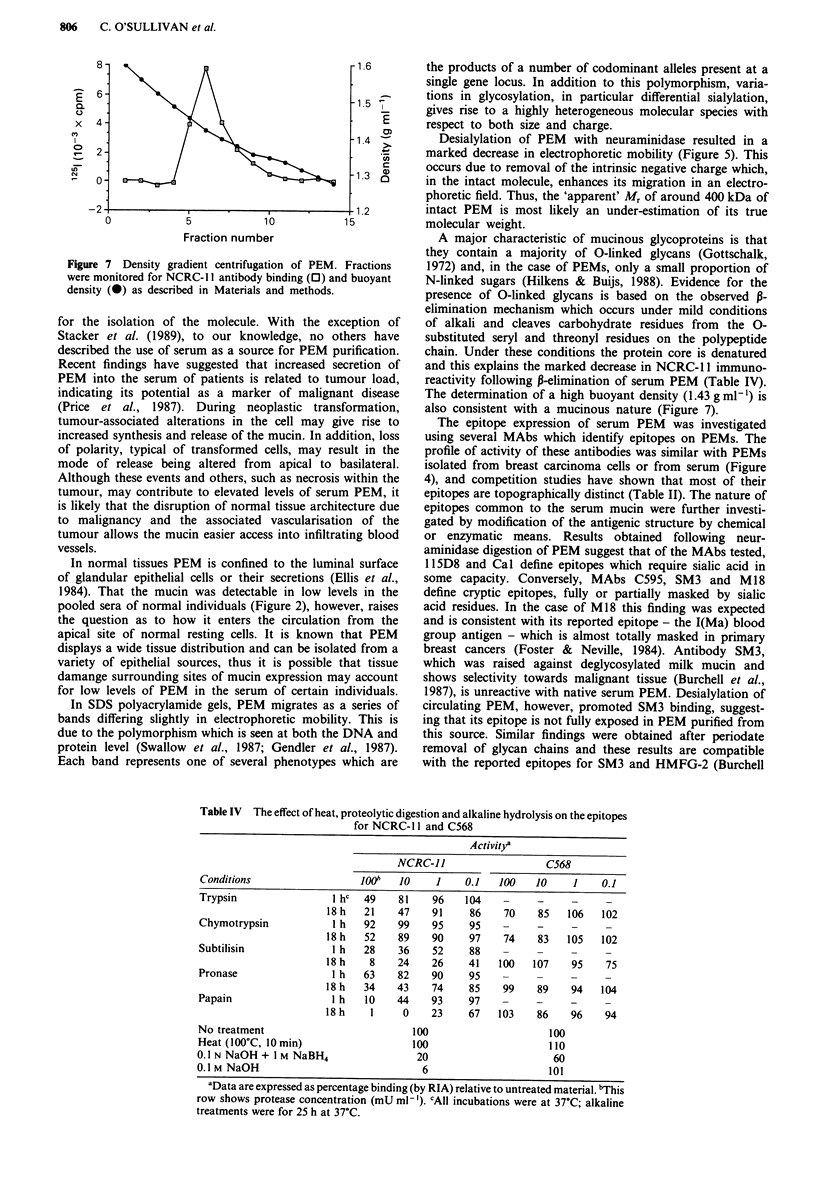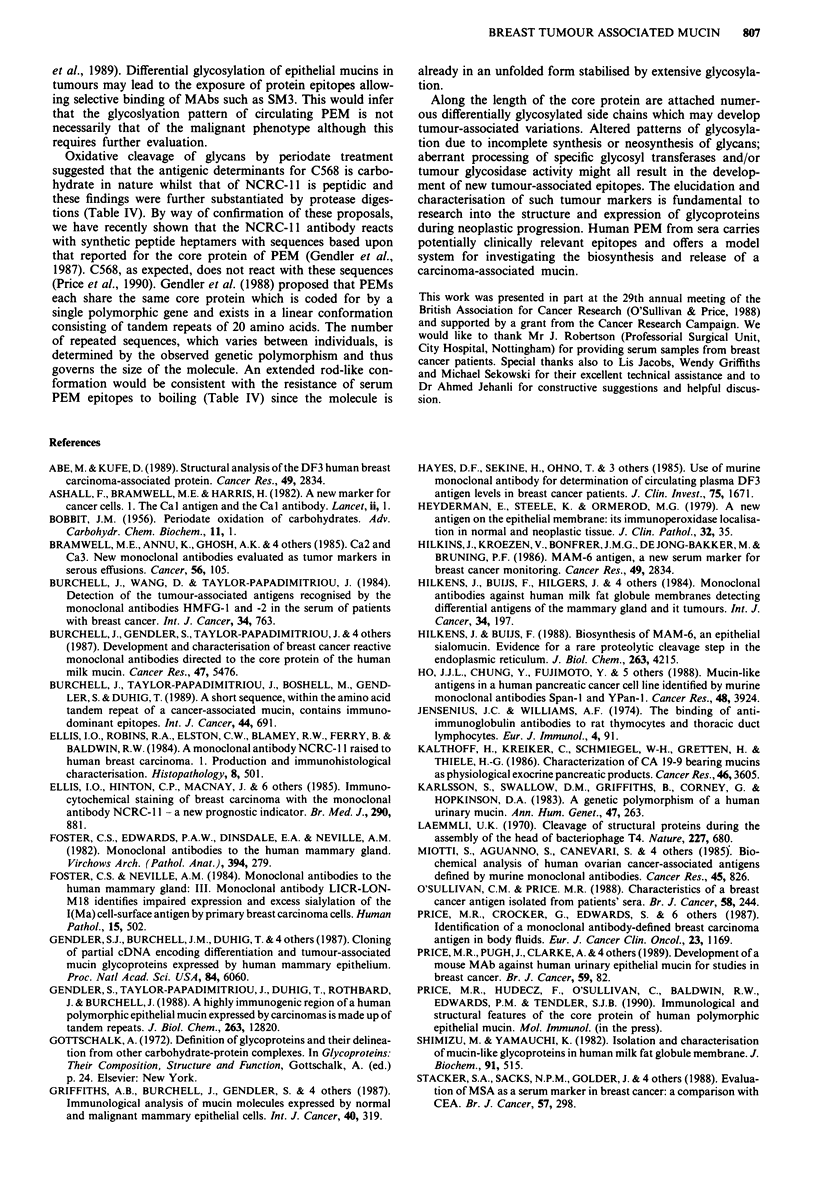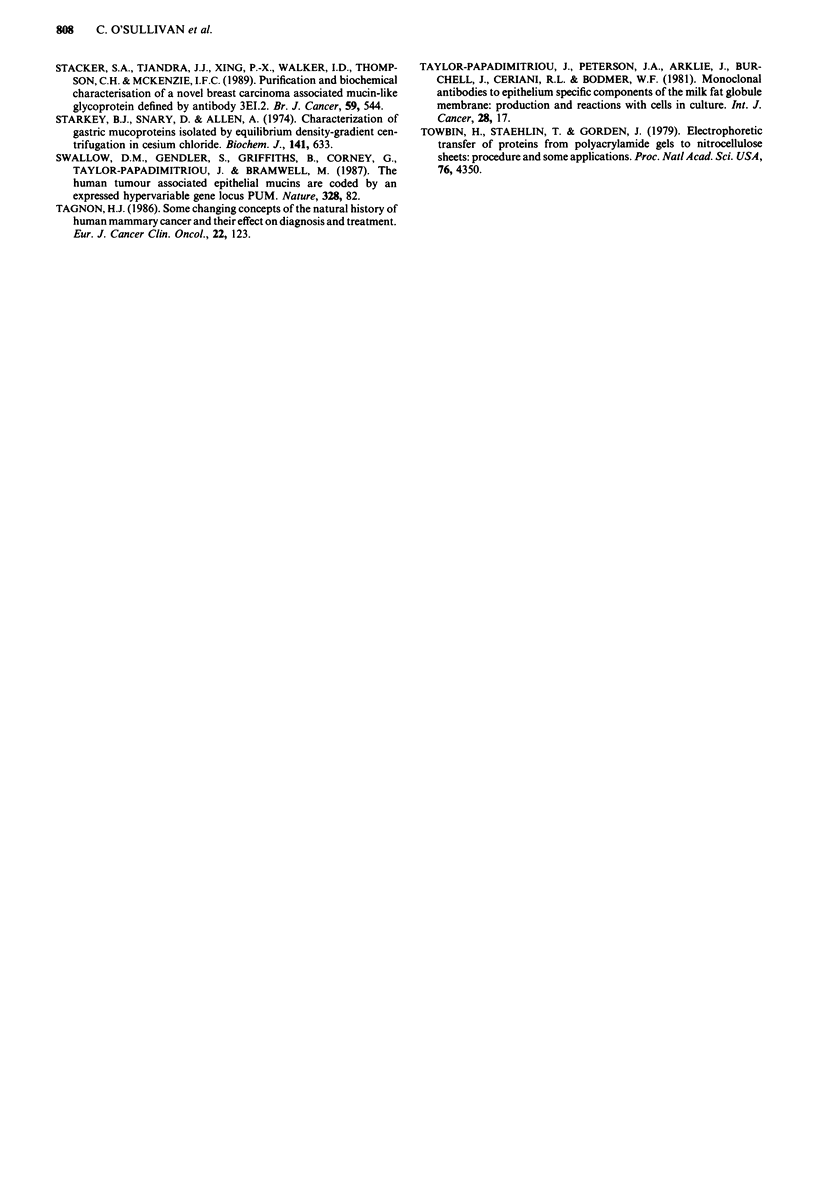# Polymorphic epithelial mucin from the sera of advanced breast cancer patients--isolation and partial characterisation.

**DOI:** 10.1038/bjc.1990.181

**Published:** 1990-06

**Authors:** C. O'Sullivan, M. R. Price, R. W. Baldwin

**Affiliations:** Cancer Research Campaign Laboratories, University of Nottingham, University Park, UK.

## Abstract

**Images:**


Br. J. Cancer (1990), 61, 801–808 © Macmillan Press Ltd., 1990

### Polymorphic epithelial mucin from the sera of advanced breast cancer patients – isolation and partial characterisation

C. O'Sullivan, M.R. Price & R.W. Baldwin

*Cancer Research Campaign Laboratories, University of Nottingham, University Park, Nottingham NG7 2RD, UK.*

**Summary** The anti-breast carcinoma monoclonal antibody (MAb), NCRC-11 defines a polymorphic epithelial mucin (PEM) which is elevated in the circulation of advanced breast carcinoma patients. Here we describe the purification and partial characterisation of this component from patients' sera and its use in the production of a second generation MAb, C568 (IgM). Pooled sera was fractionated by immunoaffinity and size-exclusion chromatography and the purity of preparations assessed by sodium dodecyl sulphate polyacrylamide gel electrophoresis (SDS-PAGE) and immunoblotting. Serum-derived PEM shows a similar pattern of electrophoretic mobility to PEM isolated from primary breast tumour tissue and migrates as several bands in 4% SDS polyacrylamide gels ($M_r > 400,000$). The epitope expression of PEMs isolated from either source is also similar, with both bearing topographically distinct determinants for several anti-mucin MAbs. The immunoreactivities of antibodies C568 and NCRC-11 were unaffected by boiling, reduction and alkylation, or by enzyme desialylation of PEM. Periodate oxidation and proteolytic digestion have suggested that the antigenic determinant for C568 is carbohydrate in nature whilst that of NCRC-11 is peptidic. In accord with the mucinous nature of the molecule, serum-derived PEM is susceptible to reductive $\beta$-elimination, elutes in the void volume of a Sepharose CL-4B column and has a buoyant density of 1.45 g ml$^{-1}$.

In Western countries breast carcinoma affects an estimated one in fourteen women and is responsible for the highest female mortality rate due to any form of malignancy (Tagnon, 1986). Consequently, the immunological detection of a carcinoma-associated marker which differentiates normal from malignant cells has become increasingly important. Since the advent of monoclonal antibody (MAb) technology, a considerable number of antibodies have been described which recognise breast carcinoma-associated antigens. These antigens show marked variations in molecular weight and tissue distribution and consequently, the antibodies defining them vary widely in their selectivity towards malignant tissue. Several of these antibodies define epitopes on a high molecular weight mucin which is peculiar to the luminal surface of glandular epithelial cells or their secretions and hence is also associated with certain carcinomas. Originally purified from human milk, this component has been termed PAS-O by Shimizu and Yamauchi (1982), EMA (epithelial membrane antigen) by Heyderman *et al*., (1979), MAM-6 by Hilkens *et al*. (1984) and MSA (mammary serum antigen) by Stacker *et al*. (1988). Karlsson *et al*. (1983) first reported that the mucin exhibits a genetic polymorphism which was later shown to exist at both the protein and DNA level (Swallow *et al*., 1987; Gendler *et al*., 1987, respectively). Consequently, Gendler *et al*., (1988) have named the mucin polymorphic epithelial mucin, or PEM, a term which aptly describes its properties.

NCRC-11 is a murine MAb (IgM) originally produced against metastatic breast carcinoma cells (Ellis *et al*., 1984). This antibody reacts with a variety of normal and malignant tissues of epithelial origin and immunocytochemical studies have shown that in malignant tissue there exists a direct relationship between the intensity of staining and patient survival (Ellis *et al*., 1985). The antigen defined by NCRC-11 bears multiple epitopes for several anti-mucin MAbs and also displays the same molecular weight, tissue distribution and genetic polymorphism as PEM. Furthermore, NCRC-11 has recently been shown to react with the core protein of PEM confirming the notion that the NCRC-11 defined antigen is indeed PEM (Price *et al*., 1990).

Of clinical interest, it has been reported that PEMs are elevated in the circulation of advanced breast carcinoma patients (Burchell *et al*., 1984; Hayes *et al*., 1985; Hilkens *et al*., 1986; Price *et al*., 1987) and these findings have promoted investigation into its potential as a prognostic or diagnostic indicator. The present study describes the purification and partial characterisation of PEM isolated from advanced breast carcinoma patients' sera and its use in the production of a second-generation MAb, C568.

Correspondence: C. O'Sullivan.
Received 16 August 1989; and in revised form 23 November 1989.

### Materials and methods

### *Monoclonal antibodies*

NCRC-11 monoclonal antibody (IgM) was raised against dissociated breast carcinoma cells as described in detail by Ellis *et al*. (1984).

MAb C568 (IgM) was derived from a fusion of mouse myeloma cells with spleen cells of a BALB/c mouse immunised against PEM isolated from sera. Hybridoma supernatants were screened by radioimmunoassay (RIA) for reactivity against purified serum PEM and positive clones selected for on this basis. Both antibodies were purified from ascitic fluids by Sepharose-Lentil lectin affinity chromatography (Pharmacia, Uppsala, Sweden).

Other MAbs used in this study and which also identify mucinous antigens are listed in Table I.

### *Solid phase RIA for PEM detection*

PEM was added to microtest plates (Falcon 3034F Terasaki plates, Becton Dickinson, CA, USA) at 10 µl per well and air-dried. Non-specific adsorption sites were blocked by incubation (30 min) with phosphate buffered saline (PBS) containing 0.1% casein (Sigma Chemical Co., Poole, Dorset). Wells were then incubated (1–2 h, room temperature) with 10 µl of the relevant MAb (or control) diluted in casein buffer. Plates were washed four times with buffer and then incubated (1 h, room temperature) with 10 µl $^{125}$I-labelled F(ab')$_2$ fragments of rabbit anti-mouse Ig (10$^5$ c.p.m. per well). After washing six times, the radioactivity retained in each well was determined. Each test was performed in triplicate and the mean ± s.d. recorded.

In direct antibody binding assays, PEM adsorbed to plates was detected directly by incubation with radiolabelled NCRC-11 antibody. Human IgM and IgG were detected using radiolabelled rabbit anti-human IgM (Dako Ltd, High Wycombe, Bucks.) and Protein A (Pharmacia), respectively. In competitive inhibition studies, the inhibiting antibody or

**Table I** MAbs to PEMs available for comparative studies

| *MAb* | *Class/ subclass* | *Immunogen* | *Ref.* |
|---|---|---|---|
| NCRC-11 | IgM | Metastatic breast cancer | Ellis *et al.* (1984) |
| C568 | IgM | PEM from breast carcinoma patients' sera | Present study |
| C595 | IgG3 | Affinity purified urinary PEM | Price *et al.* (1989) |
| SM3 | IgG1 | Deglycosylated milk mucin | Burchell *et al.* (1987) |
| Ca1 | IgM | Laryngeal carcinoma (Hep 2 cells) | Ashall *et al.* (1982) |
| Ca2 | IgG1 | Ca1 antigen | Bramwell *et al.* (1985) |
| Ca3 | IgG1 | Desialylated Ca1 antigen | Bramwell *et al.* (1985) |
| HMFG-1 | IgG1 | Human milk fat globule | Taylor-Papadimitriou *et al.* (1981) |
| HMFG-2 | IgG1 | Human milk fat globule | Taylor-Papadimitriou *et al.* (1981) |
| 115D8 | IgG1 | Human milk fat globule | Hilkens *et al.* (1984) |
| 115F5 | IgG1 | Human milk fat globule | Hilkens *et al.* (1984) |
| 115G2 | IgG2 | Human milk fat globule | Hilkens *et al.* (1984) |
| M8 | IgG1 | Human milk fat globule | Foster *et al.* (1982) |
| M18 | IgM | Human milk fat globule | Foster *et al.* (1982) |
| M24 | IgM | Human milk fat globule | Foster *et al.* (1982) |

buffer alone was admixed with equal volumes of a fixed amount of radiolabelled NCRC-11 antibody and incubated with PEM-coated plates for 1–2 h at room temperature. One hundred per cent binding was taken as the radioactivity bound using buffer alone. All reagents were labelled with $^{125}I$ (Amersham International, Bucks.) at 20 $\mu Ci\ \mu g^{-1}$ using the chloramine T method of Jensenius and Williams (1974).

### *SDS-polyacrylamide gel electrophoresis*

SDS-PAGE was performed on an LKB Midget Gel apparatus essentially according to the method of Laemmli (1970) and using either 4% or 7.5% running gels with 3% stackers. Samples were heated at 100°C for 5 min (under reducing or non-reducing conditions) before electrophoresis. After separation, proteins were stained with 0.25% Coomassie Brilliant Blue R-250 (Sigma) or transferred on to nitrocellulose membranes by Western blotting.

### *Immunoblotting*

After electrophoresis proteins were electrophoretically transferred on to nitrocellulose membranes (Bio-Rad Laboratories Ltd, Watford, Herts.) following the procedure of Towbin *et al.* (1979). Immunostaining of serum PEM was achieved by incubating the membrane with the following solutions at room temperature: (a) 0.1% casein in PBS (1 h); (b) NCRC-11 antibody (1–5 $\mu g\ ml^{-1}$) in casein buffer containing 2% normal rabbit serum (1–2 h); (c) horseradish peroxidase-conjugated rabbit anti-mouse Ig (Dako) (1 h). Between incubations the membranes were washed several times with casein buffer. Finally, bound antibodies were detected using the peroxidase substrate of 0.4% 3-amino-9-ethyl carbazole/ 0.025% $H_2O_2$. Detection of contaminating human immunoglobulins (IgG and IgM) was achieved using peroxidase-conjugated rabbit anti-human IgG and -IgM respectively (Dako). It was possible to probe blots simultaneously with more than one specific immunoconjugate without loss of staining resolution (Figure 1, for example).

### *Isolation of circulating PEM*

An immunoadsorbent column was prepared by coupling 20 mg NCRC-11 antibody to 20 ml CNBr-activated Sepharose (Pharmacia). For each purification, 50–100 ml of pooled advanced breast cancer patients' sera was filtered, clarified (180,000 *g*, 1 h) and delipidated. The sera were diluted 10-fold in 0.1 M Tris-HCl (pH 7.6) then passaged through the affinity column which had been equilibrated in the same buffer. The column was then washed with buffer and non-specifically bound material removed with a wash containing 1 M NaCl. Bound material was eluted using 0.1 M diethylamine (pH 11.5) and eluted fractions (2 ml) neutralised with 1 M Tris-HCl (pH 7.6). Ten $\mu l$ aliquots of each fraction were then assayed for PEM activity by Solid Phase RIA.

**Figure 1** SDS-PAGE Western blot analysis of isolated PEM from breast cancer patients' sera. Steps involved in the identification and elimination of contaminating human immunoglobulins are shown. 7.5% gel, non-reducing conditions. The blot was probed simultaneously, using three separate labels, for PEM, human IgM and human IgG (details in Materials and methods). Track 1, PEM after gel filtration of reduced and alkylated affinity purified material; track 2, PEM partially purified by affinity chromatography (contaminating immunoglobulins are indicated by arrows); track 3, human IgM standard; track 4, human IgG standard. The molecular weight markers are myosin heavy chain (200 kDa), phosphorylase b (92 kDa) and albumin (67 kDa).

After dialysis against PBS, pooled fractions containing PEM were concentrated, reduced with 50 mM dithiothreitol (30 min, room temperature) and subsequently alkylated with 75 mM iodoacetamide (1 h, room temperature, in the dark). The sample was then chromatographed on a 113 × 1.6 cm column of S300 gel equilibrated in PBS/0.02% $NaN_3$ and 2 ml fractions collected and assayed for PEM activity. Fractions containing PEM were pooled, concentrated and assessed for purity by SDS-PAGE. Due to their high level of glycosylation, mucins fail to stain with most standard protein stains and, in our hands, even the PAS-silver stain failed to give reproducible results. One criterion of purity was therefore the 'absence' of contaminating bands in Coomassie stained gels.

*Density gradient ultracentrifugation*

Ultracentrifugation of serum PEM was performed in a CsCl isopycnic density gradient with a starting density of 1.43 g $ml^{-1}$. The gradient was formed following centrifugation in an MSE 6 × 16.5 swing-out rotor at 110,000 *g* for 70 h at 10°C. One ml fractions were collected from the bottom of the tube and assayed for PEM activity by RIA. The density of each fraction was determined gravimetrically using an analytical balance.

*Chemical and enzymatic treatments*

Reductive β-elimination of lyophilised PEM was performed using 0.1 M NaOH with or without 1 M $NaBH_4$. After incubation (25 h, 37°C) the solutions were neutralised with glacial acetic acid and dialysed against PBS. Mild periodate oxidation was accomplished by incubating PEM-coated plates with 0, 1, 10 and 100 mM $NaIO_4$ in acetate buffer (50 mM, pH 4.5) for 12 h at 4°C in the dark. Following a brief rinse with acetate buffer, the plates were incubated with 50 mM sodium borohydride in PBS for 30 min at room temperature. (This treatment reduces aldehyde groups to alcohols and prevents non-specific cross-linking of antibody to antigen.) Plates were then washed and assayed for PEM activity by RIA.

The following enzymatic conditions were used: *Clostridium perfringens* neuraminidase (type X) 100, 10, 0 units $ml^{-1}$ in acetate buffer (0.2 M, pH 5.5) containing 1 mM phenylmethlysulphonyl fluoride (PMSF), 2 h, 37°C; trypsin, chymotrypsin, pronase E (protease, type XIV, pre-heated 60°C, 1 h), subtilisin (protease, type XXIV) and papain (containing 5 mM L-cysteine), all at 100, 10, 1, 0.1 and 0 units $ml^{-1}$ in 0.1 M Tris-HCl (pH 8.0) containing 1 mM $CaCl_2$ and incubated for 1 h and 18 h at 37°C. All enzymes were obtained from the Sigma Chemical Co.

### Results

*Purification of circulating PEM*

Pooled advanced breast cancer patients' sera was applied to an NCRC-11 affinity column. Fractions eluted from the column and assayed for PEM activity (Figure 2) established that recoverable levels of circulating PEM were exhausted after 4–6 sequential passages through the column. SDS-PAGE and Western blot analyses of affinity purified material revealed two contaminating proteins in the preparation (Figure 1, track 2) which were tentatively identified as IgG and IgM. These were confirmed to be human antibodies by running the corresponding normal standards (non-reduced human IgG and IgM) and by immunostaining blots using specific immunoconjugates for human Ig's (Figure 1, tracks 2–4). We have since established that these antibodies are normal heterophile antibodies cross-reactive with the murine NCRC-11 antibody bound to the column (O'Sullivan *et al.*, in preparation).

In order to remove these contaminants, size exclusion chromatography using Sepharose CL-4B was performed on the affinity purified material. PEM elutes coincident with the void volume indicating an apparent molecular weight in excess of 2,000 kDa. This unexpectedly high molecular weight elution profile is most likely the result of aggregation of individual molecules and has also been described by other workers (Ho *et al.*, 1988; Miotti *et al.*, 1985; Kalthoff *et al.*, 1986). Although the majority of contaminating antibody was eliminated by this means, a proportion of aggregated IgM co-eluted with the mucin. However, subsequent experiments have shown that, by reducing the contaminating IgM into its monomeric sub-units, it could be displaced into the included volume of an S300 gel filtration column thus separating it from the peak of PEM activity which remains in the void volume in S300 gel (as with CL-4B). This was achieved by reduction and alkylation of affinity purified mucin preparations prior to size exclusion chromatography. Individual fractions eluting from the S300 column were assayed separately, by RIAs, for the presence of PEM, human IgG and human IgM using the appropriate radiolabels. Figure 3 confirms the virtual exclusion of all human immunoglobulin by this method. Likewise, SDS-PAGE Western blot analysis revealed the successful removal of previously contaminating bands (Figure 1, track 1). Reduction and alkylation of PEM did not affect either its antigenicity or electrophoretic mobility. Furthermore, the presence of SDS or sulphydryl reducing agents (β-mercaptoethanol) in gel electrophoresis had no apparent effect on the molecule. These findings are consistent with the typically low levels of sulphur-containing amino acids found in PEMs (Schimizu & Yamauchi, 1982, Burchell *et al.*, 1987; Abe & Kufe, 1989). Furthermore, no evidence for intermolecular disulphide bonds which link mucin multimers has been observed in this class of molecule (Hilkens & Buijs, 1988).

In order to compare the level of circulating PEM found in the sera of advanced breast cancer patients with that found in healthy individuals, a similar fractionation of age-matched normal human sera was undertaken. Fractions eluted from the affinity column also revealed the presence of circulating PEM, albeit in relatively trace amounts (Figure 2).

*Epitope expression of PEMs*

A number of MAbs (see Table I) raised against various immunogens and known to be reactive with PEMs isolated from breast carcinoma cells were tested, by RIA, for their reactivity against PEMs isolated from both breast carcinoma cells and patients' sera. Figure 4 demonstates that the overall profile of antibody reactivity is similar for antigen isolated from either source. Clearly, the antigenic determinant for

**Figure 2** Immunoadsorbent purification of PEM from pooled breast carcinoma patients' sera (●) compared to a similar fractionation of pooled normal sera (□). Elution was achieved using 0.1 M diethylamine (pH 11.5) and individual fractions were assayed for PEM activity by RIA.

**Figure 3** Removal of human immunoglobulin from reduced and alkylated affinity purified PEM by size exclusion chromatography. Fractions (1.6 ml) eluting from an S300 gel filtration column were assayed, by RIAs, for PEM, human IgG and human IgM using $^{125}$I-labelled NCRC-11 antibody (●), $^{125}$I-labelled protein A (▲) and $^{125}$I-labelled anti-human IgM (O) respectively. The exclusion volume of the column (A) and elution points of ferritin (b, 440 kDa), IgG (C, 155 kDa) and albumin (D, 67 kDa) are indicated by arrows.

**Figure 4** Comparative epitope expression of PEM using the anti-PEM MAbs listed in Table I. PEMs isolated from breast carcinoma cells (□) and breast cancer patients' sera (■) were adsorbed to the wells of microtest plates and analysed for antibody binding by RIA.

each of the MAbs tested is neither lost nor denatured in the circulating mucin either before or after its release from the epithelial cell.

Competitive inhibition studies using $^{125}$I-labelled NCRC-11 antibody were performed on serum antigen preparations to ascertain if any of the anti-PEM MAbs recognise the same, or a topographically related epitope as NCRC-11 MAb. Table II shows that C568 and NCRC-11 MAbs define separate and distinct epitopes since no inhibition was observed even at the highest concentration of C568. Of the MAbs tested, only M8, HMFG-2 and Ca2 inhibited NCRC-11 binding to any significant extent with this most marked in the case of the anti-milk fat globule MAb, M8. Thus, these antibodies either share the same epitope as NCRC-11 or their epitopes are in close enough proximity to cause mutual inhibition due to steric effects. The latter is most likely in the case of Ca2 and HMFG-2 where only around 50% inhibition was observed at the highest concentration of competing antibody compared to 86% inhibition in the case of MAb M8 (although these tests do not establish true identity between epitopes). No inhibition was observed with any of the other MAbs tested suggesting that they define separate antigenic determinants to that recognised by NCRC-11. Inefficient radiolabelling of C568, resulting in a marked loss of immunoreactivity, has so far prevented a reciprocal study on the epitope expression of C568 in comparison to other PEM reactive MAbs.

### *Neuraminidase treatment of PEM*

On 4% polyacrylamide gels PEM migrates as a series of bands which correspond to an apparent $M_r$ of >400,000 (Figure 5, track 2). Following neuraminidase treatment of serum PEM, a marked decrease in electrophoretic mobility was observed, with the molecule barely entering the gel (Figure 5, track 1). No apparent change in antigenicity was observed although there was a loss in resolution of the phenotypic bands. To investigate the role of sialic acid residues in relation to individual epitope structures, epitope expression studies on desialylated PEM were undertaken.

**Figure 5** Western blot anlysis of neuraminidase treated PEM. Purified serum PEM was subjected to neuraminidase digestion as described in Materials and methods. Digested and undigested samples were electrophoresed on a 4% polyacrylamide gel and then transferred on to nitrocellulose. After blocking, the nitrocellulose was incubated with NCRC-11 antibody followed by peroxidase conjugated anti-mouse Ig and developed with the peroxidase substrate 3-amino-9-ethyl carbazole/$H_2O_2$. Track 1 neuraminidase treated PEM; track 2, untreated PEM.

**Table II** Competitive inhibition of $^{125}$I-NCRC-11 antibody binding to purified serum PEM

| | *Percentage binding of $^{125}$I-labelled NCRC-11 in the presence of* | | | | | | | |
|---|---|---|---|---|---|---|---|---|
| *Hybridoma tissue culture supernatants*[a] | | | | | | | | |
| *Dilution*[c] | *NCRC-11* | *C568* | *HMFG-1* | *HMFG-2* | *Ca1* | *Ca2* | | |
| $10^{0}$ | 1 | 94 | 87 | 42 | 104 | 56 | | |
| $10^{-1}$ | 19 | 121 | 96 | 83 | 105 | 86 | | |
| $10^{-2}$ | 81 | 113 | 103 | 90 | 108 | 105 | | |
| $10^{-3}$ | 92 | 112 | 97 | 99 | 98 | 112 | | |
| *Hybridoma ascitic fluids*[b] | | | | | | | | |
| *Dilution* | *NCRC-11* | *C568* | *M8* | *M18* | *115D8* | *115F5* | *115G2* | *NIgM* |
| $10^{-3}$ | 0 | 92 | 14 | 84 | 103 | 79 | 95 | 118 |
| $10^{-4}$ | 23 | 85 | 31 | 91 | 109 | 72 | 123 | 106 |
| $10^{-5}$ | 78 | 93 | 89 | 106 | 130 | 91 | 116 | 106 |
| $10^{-6}$ | 109 | 102 | 85 | 100 | 128 | 84 | 106 | 95 |

Monoclonal antibodies in [a]tissue culture supernatants and [b]ascitic fluids were tested for their capacity to inhibit the binding of radiolabelled NCRC-11 antibody at the dilutions shown. [c]Dilutions of competing antibodies were predetermined to ensure saturation of the antigen at least at the highest concentration used.

**Table III** MAb activities to neuraminidase treated serum PEM

| *MAb* | *Activity (% binding of control at following concentrations of neuraminidase (mU ml$^{-1}$)*[a]*)* 0 | 10 | 100 |
|---|---|---|---|
| NCRC-11 | 100 | 98 | 91 |
| C568 | 100 | 106 | 118 |
| C595 | 100 | 622 | 599 |
| Ca1 | 100 | 35 | 29 |
| Ca2 | 100 | 208 | 218 |
| Ca3 | 100 | 126 | 135 |
| M8 | 100 | 150 | 149 |
| M18 | 100 | 385 | 380 |
| 115D8 | 100 | 24 | 21 |
| 115F5 | 100 | 98 | 116 |
| 115G2 | 100 | 95 | 106 |
| SM3 | – | gains activity | gains activity |

[a]Incubations were for 2 h at 37°C after which treated and untreated mucins were tested for retention of MAb binding by RIA.

Table III shows the percentage binding retained of each antibody relative to untreated material. These results suggest that sialic acid is not required for maintaining the full immunoreactivities of MAbs C568 or NCRC-11. Clearly their epitopes, and those of 115F5 and 115G2, reside within the underlying sugar residues or on the protein core. Desialylation of PEM led to a marked reduction in the binding activities of MAbs Ca1 and 115D8 suggesting that sialic acids form part of their respective epitopes or are involved in the conformational presentation of the epitope to its target antibody. Partial retention of binding suggests that other sugar residues, or the protein moiety itself, also participate in the epitope structure. Several MAbs showed an increased reactivity towards desialylated PEM indicating that they define cryptic epitopes, masked in the native molecule by the high degree of sialylation. MAb C595 (Price *et al.*, 1989), which was raised against urinary PEM, binds at relatively low levels to serum PEM compared to its high reactivity with the urinary mucin. However, after desialylation of serum PEM a 6-fold increase in binding was observed. Similar results were obtained for MAb M18 which displayed a 3–4-fold increase in binding. The immunoreactivity of Ca2 was also enhanced after neuraminidase treatment of the antigen, and MAb SM3, which is unreactive to native serum PEM, gained activity following antigen desialylation.

### *Periodate oxidation of serum PEM*

Mild periodate oxidation of mucins at acidic pH has been shown to cleave carbohydrate vicinal hydroxyl groups without altering the structure of the polypeptide chain (Bobbit, 1956). In such a manner it is possible to destroy carbohydrate epitopes leaving intact only those epitopes common to the protein moiety and hence establish the nature of PEM epitopes. Figure 6 represents the effect oxidative cleavage of PEM has on the binding of several MAbs relative to their activity towards untreated material. The binding of MAb C568 to treated antigen was virtually abolished except at the lowest concentration of periodate used (1 mM). Likewise, 115D8 reactivity was markedly reduced. Conversely, SM3, which is unreactive with native serum-derived PEM, gained binding activity upon periodate treatment of the mucin. MAbs M8 and HMFG-2 also showed some increase in binding at the lower concentrations of periodate. The epitopes identified by these antibodies involve the protein moiety (Griffiths *et al.*, 1987) which suggests that oxidative cleavage of glycan residues further exposes their target epitopes. The binding of NCRC-11 MAb was unaffected by periodate treatment except for some decrease in activity at the highest concentration. However, at high concentrations of periodate over long incubation times, non-specific oxidation of the polypeptide chain can occur resulting in partial destruction of protein epitopes.

These results suggest that the epitope of C568 resides on the carbohydrate moiety of the molecule whilst that of NCRC-11, like M8 and HMFG-2, involves protein. It should

**Figure 6** Periodate oxidation of PEM isolated from serum. Samples were incubated (12 h, 4°C) with 100 mM (▨), 10 mM (□), and 1 mM (▩) sodium periodate in 50 mM acetate buffer (pH 4.5). After reduction with 50 mM sodium borohydride, treated or untreated (■) samples were tested for loss or gain of antibody binding using several anti-mucin MAbS.

be noted, however, that periodate oxidation cleaves only those saccharides with vicinal hydroxyls, therefore results from periodate treatment alone should be interpreted with caution since it is possible that certain epitopes may reside upon periodate-resistant sugars (e.g 1→3 linked Gal).

### *Buoyant density of PEM*

Well-documented evidence reports that mucins exhibit higher densities than other proteins and glycoproteins, usually in the range of 1.4–1.5 g ml$^{-1}$ (Starkey *et al.*, 1974). Figure 7 shows fractions recovered after CsCl density gradient centrifugation of serum PEM which were assayed for PEM activity after determination of their density. PEM was recovered in a band corresponding to a density of 1.40–1.45 g ml$^{-1}$ with the peak occuring around 1.43 g ml$^{-1}$. This is in accord with the expected value for a mucinous protein.

### *Reductive β-elimination and protease digestion of purified serum antigen*

Alkaline hydrolysis of PEM under reducing (0.1 N NaOH + 1 M $NaBH_4$) or non-reducing conditions (0.1 N NaOH) substantially decreased the immunoreactivity of NCRC-11 antibody but had little effect on that of C568 (Table IV). Proteolytic digestions using the proteases and conditions described in Table IV also had little effect on C568 activity even under exhaustive conditions (18 h) or high protease concentrations (100 mU ml$^{-1}$). In contrast, all of the proteases listed, at least at the most extreme of conditions, decreased NCRC-11 immunoreactivity by varying degrees. This effect was most pronounced in the case of proteases sublitisin and papain.

### Discussion

Significantly elevated levels of PEM in the circulation of advanced breast cancer patients provides a convenient source

**Figure 7** Density gradient centrifugation of PEM. Fractions were monitored for NCRC-11 antibody binding (□) and buoyant density (●) as described in Materials and methods.

for the isolation of the molecule. With the exception of Stacker *et al.* (1989), to our knowledge, no others have described the use of serum as a source for PEM purification. Recent findings have suggested that increased secretion of PEM into the serum of patients is related to tumour load, indicating its potential as a marker of malignant disease (Price *et al.*, 1987). During neoplastic transformation, tumour-associated alterations in the cell may give rise to increased synthesis and release of the mucin. In addition, loss of polarity, typical of transformed cells, may result in the mode of release being altered from apical to basilateral. Although these events and others, such as necrosis within the tumour, may contribute to elevated levels of serum PEM, it is likely that the disruption of normal tissue architecture due to malignancy and the associated vascularisation of the tumour allows the mucin easier access into infiltrating blood vessels.

In normal tissues PEM is confined to the luminal surface of glandular epithelial cells or their secretions (Ellis *et al.*, 1984). That the mucin was detectable in low levels in the pooled sera of normal individuals (Figure 2), however, raises the question as to how it enters the circulation from the apical site of normal resting cells. It is known that PEM displays a wide tissue distribution and can be isolated from a variety of epithelial sources, thus it is possible that tissue damange surrounding sites of mucin expression may account for low levels of PEM in the serum of certain individuals.

In SDS polyacrylamide gels, PEM migrates as a series of bands differing slightly in electrophoretic mobility. This is due to the polymorphism which is seen at both the DNA and protein level (Swallow *et al.*, 1987; Gendler *et al.*, 1987). Each band represents one of several phenotypes which are the products of a number of codominant alleles present at a single gene locus. In addition to this polymorphism, variations in glycosylation, in particular differential sialylation, gives rise to a highly heterogeneous molecular species with respect to both size and charge.

Desialylation of PEM with neuraminidase resulted in a marked decrease in electrophoretic mobility (Figure 5). This occurs due to removal of the intrinsic negative charge which, in the intact molecule, enhances its migration in an electrophoretic field. Thus, the 'apparent' $M_r$ of around 400 kDa of intact PEM is most likely an under-estimation of its true molecular weight.

A major characteristic of mucinous glycoproteins is that they contain a majority of O-linked glycans (Gottschalk, 1972) and, in the case of PEMs, only a small proportion of N-linked sugars (Hilkens & Buijs, 1988). Evidence for the presence of O-linked glycans is based on the observed $\beta$-elimination mechanism which occurs under mild conditions of alkali and cleaves carbohydrate residues from the O-substituted seryl and threonyl residues on the polypeptide chain. Under these conditions the protein core is denatured and this explains the marked decrease in NCRC-11 immunoreactivity following $\beta$-elimination of serum PEM (Table IV). The determination of a high buoyant density (1.43 g ml$^{-1}$) is also consistent with a mucinous nature (Figure 7).

The epitope expression of serum PEM was investigated using several MAbs which identify epitopes on PEMs. The profile of activity of these antibodies was similar with PEMs isolated from breast carcinoma cells or from serum (Figure 4), and competition studies have shown that most of their epitopes are topographically distinct (Table II). The nature of epitopes common to the serum mucin were further investigated by modification of the antigenic structure by chemical or enzymatic means. Results obtained following neuraminidase digestion of PEM suggest that of the MAbs tested, 115D8 and Ca1 define epitopes which require sialic acid in some capacity. Conversely, MAbs C595, SM3 and M18 define cryptic epitopes, fully or partially masked by sialic acid residues. In the case of M18 this finding was expected and is consistent with its reported epitope – the I(Ma) blood group antigen – which is almost totally masked in primary breast cancers (Foster & Neville, 1984). Antibody SM3, which was raised against deglycosylated milk mucin and shows selectivity towards malignant tissue (Burchell *et al.*, 1987), is unreactive with native serum PEM. Desialylation of circulating PEM, however, promoted SM3 binding, suggesting that its epitope is not fully exposed in PEM purified from this source. Similar findings were obtained after periodate removal of glycan chains and these results are compatible with the reported epitopes for SM3 and HMFG-2 (Burchell

**Table IV** The effect of heat, proteolytic digestion and alkaline hydrolysis on the epitopes for NCRC-11 and C568

| | | *Activity*[a] | | | | | | | |
|---|---|---|---|---|---|---|---|---|---|
| | | *NCRC-11* | | | | *C568* | | | |
| *Conditions* | | *100*[b] | *10* | *1* | *0.1* | *100* | *10* | *1* | *0.1* |
| Trypsin | 1 h[c] | 49 | 81 | 96 | 104 | – | – | – | – |
| | 18 h | 21 | 47 | 91 | 86 | 70 | 85 | 106 | 102 |
| Chymotrypsin | 1 h | 92 | 99 | 95 | 95 | – | – | – | – |
| | 18 h | 52 | 89 | 90 | 97 | 74 | 83 | 105 | 102 |
| Subtilisin | 1 h | 28 | 36 | 52 | 88 | – | – | – | – |
| | 18 h | 8 | 24 | 26 | 41 | 100 | 107 | 95 | 75 |
| Pronase | 1 h | 63 | 82 | 90 | 95 | – | – | – | – |
| | 18 h | 34 | 43 | 74 | 85 | 99 | 89 | 94 | 104 |
| Papain | 1 h | 10 | 44 | 93 | 97 | – | – | – | – |
| | 18 h | 1 | 0 | 23 | 67 | 103 | 86 | 96 | 94 |
| No treatment | | | | 100 | | | | 100 | |
| Heat (100°C, 10 min) | | | | 100 | | | | 110 | |
| 0.1 N NaOH + 1 M $NaBH_4$ | | | | 20 | | | | 60 | |
| 0.1 M NaOH | | | | 6 | | | | 101 | |

[a]Data are expressed as percentage binding (by RIA) relative to untreated material. [b]This row shows protease concentration (mU ml$^{-1}$). [c]All incubations were at 37°C; alkaline treatments were for 25 h at 37°C.

*et al.*, 1989). Differential glycosylation of epithelial mucins in tumours may lead to the exposure of protein epitopes allowing selective binding of MAbs such as SM3. This would infer that the glycoslyation pattern of circulating PEM is not necessarily that of the malignant phenotype although this requires further evaluation.

Oxidative cleavage of glycans by periodate treatment suggested that the antigenic determinants for C568 is carbohydrate in nature whilst that of NCRC-11 is peptidic and these findings were further substantiated by protease digestions (Table IV). By way of confirmation of these proposals, we have recently shown that the NCRC-11 antibody reacts with synthetic peptide heptamers with sequences based upon that reported for the core protein of PEM (Gendler *et al.*, 1987). C568, as expected, does not react with these sequences (Price *et al.*, 1990). Gendler *et al.* (1988) proposed that PEMs each share the same core protein which is coded for by a single polymorphic gene and exists in a linear conformation consisting of tandem repeats of 20 amino acids. The number of repeated sequences, which varies between individuals, is determined by the observed genetic polymorphism and thus governs the size of the molecule. An extended rod-like conformation would be consistent with the resistance of serum PEM epitopes to boiling (Table IV) since the molecule is already in an unfolded form stabilised by extensive glycosylation.

Along the length of the core protein are attached numerous differentially glycosylated side chains which may develop tumour-associated variations. Altered patterns of glycosylation due to incomplete synthesis or neosynthesis of glycans; aberrant processing of specific glycosyl transferases and/or tumour glycosidase activity might all result in the development of new tumour-associated epitopes. The elucidation and characterisation of such tumour markers is fundamental to research into the structure and expression of glycoproteins during neoplastic progression. Human PEM from sera carries potentially clinically relevant epitopes and offers a model system for investigating the biosynthesis and release of a carcinoma-associated mucin.

This work was presented in part at the 29th annual meeting of the British Association for Cancer Research (O'Sullivan & Price, 1988) and supported by a grant from the Cancer Research Campaign. We would like to thank Mr J. Robertson (Professorial Surgical Unit, City Hospital, Nottingham) for providing serum samples from breast cancer patients. Special thanks also to Lis Jacobs, Wendy Griffiths and Michael Sekowski for their excellent technical assistance and to Dr Ahmed Jehanli for constructive suggestions and helpful discussion.

### References

ABE, M. & KUFE, D. (1989). Structural analysis of the DF3 human breast carcinoma-associated protein. *Cancer Res.*, **49**, 2834.

ASHALL, F., BRAMWELL, M.E. & HARRIS, H. (1982). A new marker for cancer cells. 1. The Ca1 antigen and the Ca1 antibody. *Lancet*, **ii**, 1.

BOBBIT, J.M. (1956). Periodate oxidation of carbohydrates. *Adv. Carbohydr. Chem. Biochem.*, **11**, 1.

BRAMWELL, M.E., ANNU, K., GHOSH, A.K. & 4 others (1985). Ca2 and Ca3. New monoclonal antibodies evaluated as tumor markers in serous effusions. *Cancer*, **56**, 105.

BURCHELL, J., WANG, D. & TAYLOR-PAPADIMITRIOU, J. (1984). Detection of the tumour-associated antigens recognised by the monoclonal antibodies HMFG-1 and -2 in the serum of patients with breast cancer. *Int. J. Cancer*, **34**, 763.

BURCHELL, J., GENDLER, S., TAYLOR-PAPADIMITRIOU, J. & 4 others (1987). Development and characterisation of breast cancer reactive monoclonal antibodies directed to the core protein of the human milk mucin. *Cancer Res.*, **47**, 5476.

BURCHELL, J., TAYLOR-PAPADIMITRIOU, J., BOSHELL, M., GENDLER, S. & DUHIG, T. (1989). A short sequence, within the amino acid tandem repeat of a cancer-associated mucin, contains immunodominant epitopes. *Int. J. Cancer*, **44**, 691.

ELLIS, I.O., ROBINS, R.A., ELSTON, C.W., BLAMEY, R.W., FERRY, B. & BALDWIN, R.W. (1984). A monoclonal antibody NCRC-11 raised to human breast carcinoma. 1. Production and immunohistological characterisation. *Histopathology*, **8**, 501.

ELLIS, I.O., HINTON, C.P., MACNAY, J. & 6 others (1985). Immunocytochemical staining of breast carcinoma with the monoclonal antibody NCRC-11 – a new prognostic indicator. *Br. Med. J.*, **290**, 881.

FOSTER, C.S., EDWARDS, P.A.W., DINSDALE, E.A. & NEVILLE, A.M. (1982). Monoclonal antibodies to the human mammary gland. *Virchows Arch. (Pathol. Anat.)*, **394**, 279.

FOSTER, C.S. & NEVILLE, A.M. (1984). Monoclonal antibodies to the human mammary gland: III. Monoclonal antibody LICR-LON-M18 identifies impaired expression and excess sialylation of the I(Ma) cell-surface antigen by primary breast carcinoma cells. *Human Pathol.*, **15**, 502.

GENDLER, S.J., BURCHELL, J.M., DUHIG, T. & 4 others (1987). Cloning of partial cDNA encoding differentiation and tumour-associated mucin glycoproteins expressed by human mammary epithelium. *Proc. Natl Acad. Sci. USA*, **84**, 6060.

GENDLER, S., TAYLOR-PAPADIMITRIOU, J., DUHIG, T., ROTHBARD, J. & BURCHELL, J. (1988). A highly immunogenic region of a human polymorphic epithelial mucin expressed by carcinomas is made up of tandem repeats. *J. Biol. Chem.*, **263**, 12820.

GOTTSCHALK, A. (1972). Definition of glycoproteins and their delineation from other carbohydrate-protein complexes. In *Glycoproteins: Their Composition, Structure and Function*, Gottschalk, A. (ed.) p. 24. Elsevier: New York.

GRIFFITHS, A.B., BURCHELL, J., GENDLER, S. & 4 others (1987). Immunological analysis of mucin molecules expressed by normal and malignant mammary epithelial cells. *Int. J. Cancer*, **40**, 319.

HAYES, D.F., SEKINE, H., OHNO, T. & 3 others (1985). Use of murine monoclonal antibody for determination of circulating plasma DF3 antigen levels in breast cancer patients. *J. Clin. Invest.*, **75**, 1671.

HEYDERMAN, E., STEELE, K. & ORMEROD, M.G. (1979). A new antigen on the epithelial membrane: its immunoperoxidase localisation in normal and neoplastic tissue. *J. Clin. Pathol.*, **32**, 35.

HILKINS, J., KROEZEN, V., BONFRER, J.M.G., DE JONG-BAKKER, M. & BRUNING, P.F. (1986). MAM-6 antigen, a new serum marker for breast cancer monitoring. *Cancer Res.*, **49**, 2834.

HILKENS, J., BUIJS, F., HILGERS, J. & 4 others (1984). Monoclonal antibodies against human milk fat globule membranes detecting differential antigens of the mammary gland and it tumours. *Int. J. Cancer*, **34**, 197.

HILKENS, J. & BUIJS, F. (1988). Biosynthesis of MAM-6, an epithelial sialomucin. Evidence for a rare proteolytic cleavage step in the endoplasmic reticulum. *J. Biol. Chem.*, **263**, 4215.

HO, J.J.L., CHUNG, Y., FUJIMOTO, Y. & 5 others (1988). Mucin-like antigens in a human pancreatic cancer cell line identified by murine monoclonal antibodies Span-1 and YPan-1. *Cancer Res.*, **48**, 3924.

JENSENIUS, J.C. & WILLIAMS, A.F. (1974). The binding of anti-immunoglobulin antibodies to rat thymocytes and thoracic duct lymphocytes. *Eur. J. Immunol.*, **4**, 91.

KALTHOFF, H., KREIKER, C., SCHMIEGEL, W-H., GRETTEN, H. & THIELE, H.-G. (1986). Characterization of CA 19-9 bearing mucins as physiological exocrine pancreatic products. *Cancer Res.*, **46**, 3605.

KARLSSON, S., SWALLOW, D.M., GRIFFITHS, B., CORNEY, G. & HOPKINSON, D.A. (1983). A genetic polymorphism of a human urinary mucin. *Ann. Hum. Genet.*, **47**, 263.

LAEMMLI, U.K. (1970). Cleavage of structural proteins during the assembly of the head of bacteriophage T4. *Nature*, **227**, 680.

MIOTTI, S., AGUANNO, S., CANEVARI, S. & 4 others (1985). Biochemical analysis of human ovarian cancer-associated antigens defined by murine monoclonal antibodies. *Cancer Res.*, **45**, 826.

O'SULLIVAN, C.M. & PRICE. M.R. (1988). Characteristics of a breast cancer antigen isolated from patients' sera. *Br. J. Cancer*, **58**, 244.

PRICE, M.R., CROCKER, G., EDWARDS, S. & 6 others (1987). Identification of a monoclonal antibody-defined breast carcinoma antigen in body fluids. *Eur. J. Cancer Clin. Oncol.*, **23**, 1169.

PRICE, M.R., PUGH, J., CLARKE, A. & 4 others (1989). Development of a mouse MAb against human urinary epithelial mucin for studies in breast cancer. *Br. J. Cancer*, **59**, 82.

PRICE, M.R., HUDECZ, F., O'SULLIVAN, C., BALDWIN, R.W., EDWARDS, P.M. & TENDLER, S.J.B. (1990). Immunological and structural features of the core protein of human polymorphic epithelial mucin. *Mol. Immunol.* (in the press).

SHIMIZU, M. & YAMAUCHI, K. (1982). Isolation and characterisation of mucin-like glycoproteins in human milk fat globule membrane. *J. Biochem.*, **91**, 515.

STACKER, S.A., SACKS, N.P.M., GOLDER, J. & 4 others (1988). Evaluation of MSA as a serum marker in breast cancer: a comparison with CEA. *Br. J. Cancer*, **57**, 298.

STACKER, S.A., TJANDRA, J.J., XING, P.-X., WALKER, I.D., THOMPSON, C.H. & MCKENZIE, I.F.C. (1989). Purification and biochemical characterisation of a novel breast carcinoma associated mucin-like glycoprotein defined by antibody 3EI.2. *Br. J. Cancer*, **59**, 544.

STARKEY, B.J., SNARY, D. & ALLEN, A. (1974). Characterization of gastric mucoproteins isolated by equilibrium density-gradient centrifugation in cesium chloride. *Biochem. J.*, **141**, 633.

SWALLOW, D.M., GENDLER, S., GRIFFITHS, B., CORNEY, G., TAYLOR-PAPADIMITRIOU, J. & BRAMWELL, M. (1987). The human tumour associated epithelial mucins are coded by an expressed hypervariable gene locus PUM. *Nature*, **328**, 82.

TAGNON, H.J. (1986). Some changing concepts of the natural history of human mammary cancer and their effect on diagnosis and treatment. *Eur. J. Cancer Clin. Oncol.*, **22**, 123.

TAYLOR-PAPADIMITRIOU, J., PETERSON, J.A., ARKLIE, J., BURCHELL, J., CERIANI, R.L. & BODMER, W.F. (1981). Monoclonal antibodies to epithelium specific components of the milk fat globule membrane: production and reactions with cells in culture. *Int. J. Cancer*, **28**, 17.

TOWBIN, H., STAEHLIN, T. & GORDEN, J. (1979). Electrophoretic transfer of proteins from polyacrylamide gels to nitrocellulose sheets: procedure and some applications. *Proc. Natl Acad. Sci. USA*, **76**, 4350.